# Update on Cases of Delayed Hemolysis After Parenteral Artesunate Therapy for Malaria — United States, 2008 and 2013

**Published:** 2014-08-29

**Authors:** Magdalena M. Paczkowski, Keren L. Landman, Paul M. Arguin

**Affiliations:** 1 Epidemic Intelligence Service, CDC; 2 Division of Parasitic Diseases and Malaria, Center for Global Health, CDC

Parenteral artesunate, a first-line treatment for severe malaria in several countries, is associated with increased survival and has a better safety profile compared with parenteral quinine or quinidine (1,2). However, parenteral artesunate has been associated with delayed hemolysis, leading to concerns about drug toxicity. Postartemisinin delayed hemolysis (PADH) can occur 1–3 weeks after initiation of treatment with artemisinin-based antimalarials such as artesunate and is characterized by a decline in hemoglobin levels amid hemolysis. CDC conducted a literature review and identified 18 cases of PADH since 2012, mostly in European travelers. In addition, malaria case reports were reviewed retrospectively, and active surveillance was implemented in the United States, identifying two additional PADH cases, for a total of 20. A few patients with PADH required blood transfusions, but among patients where complete follow-up information was available, all made a full recovery. Results from this review suggest that PADH occurs because of delayed clearance of once-infected erythrocytes, probably as a result of a pharmacologic effect of parenteral artesunate and not drug-related toxicity. Therefore, parenteral artesunate can still be considered a safe treatment for severe malaria and should remain an option for its treatment.

Malaria, a mosquito-borne disease caused by *Plasmodium* spp., was eliminated from the United States in the 1950s. Since 1971, about 1,500 cases per year have been reported in the United States, almost all imported by travelers who went to malaria-endemic areas (3). In the United States, several antimalarials are available for treatment of uncomplicated malaria, including atovaquone-proguanil, artemether-lumefantrine, chloroquine, oral quinine plus doxycycline (or tetracycline or clindamycin), and mefloquine, depending on species and expected sensitivity.

Antimalarial options for treatment of severe malaria, however, are limited. The CDC-recommended treatment, parenteral quinidine (given with a second drug: doxycycline, tetracycline, or clindamycin), is the only Federal Drug Administration (FDA)–approved parenteral antimalarial regimen currently available in the United States. Until 1985, parenteral quinine was the only option for treatment of severe malaria even though it was not available commercially and had to be obtained through CDC. In 1991, a data review resulted in the new recommendation of parenteral quinidine as first-line treatment, and parenteral quinine became no longer accessible through CDC (4). Since 2007, parenteral artesunate has been available for treatment of severe malaria in the United States through an investigational new drug (IND) protocol with FDA (5). Currently, parenteral artesunate is only released by CDC when parenteral quinidine is contraindicated, not tolerated, or not available. Treatment of severe malaria with parenteral artesunate is associated with increased survival and fewer side effects compared with treatment with parenteral quinine or quinidine (1,2). However, delayed hemolysis has been reported in association with parenteral artesunate therapy of malaria (6–8).

In PADH, hemoglobin levels decline as a result of hemolysis 1–3 weeks following initiation of treatment of malaria with a drug in the artemisinin class of antimalarials (e.g., artesunate) (6,8). No deaths have been attributed to PADH, but levels of hemoglobin as low as 2.8 g/dL (normal ranges = men, 14.0–17.0 g/dL; women, 12.5–15.0 g/dL; and children, 10.3–16.0 g/dL) have been reported, and patients have required blood transfusions (6,7). No demographic risk factors for PADH have been identified. Multiple case definitions for PADH have been suggested, resulting in a wide range of incidence rates.

A literature review conducted in 2012 identified 19 PADH cases during 2010–2012, the majority occurring in European travelers returning from countries where malaria is endemic (6). To update this literature review, CDC conducted a search via the National Library of Medicine’s PubMed and identified 18 additional PADH cases. Keywords used included “artesunate” combined with “delayed hemolysis” and “hemolytic anemia.” Five of the 18 cases were reported in children from Gabon and Ghana, with nadir hemoglobin levels ranging from 2.8 g/dL to 8.5 g/dL, and a peak lactate dehydrogenase (LDH) value ranging from 400 U/L to 1,400 U/L (normal = 125–240 U/L) (7). In one published report, a surveillance study of returned travelers in France, 13 PADH cases were reported, with a decline of 12% in mean hemoglobin levels amid increased hemolytic activity 7–14 days after treatment initiation with artesunate (8).

As a result of the findings from the 2012 literature review, the CDC artesunate IND protocol was amended in 2012 to encourage active surveillance for PADH. As a result, for the first time since initiation of the IND protocol in 2007, two new cases in the United States, among 244 artesunate treatments administered by CDC up to August 2014, were detected by CDC. One case, occurring in 2008, was detected through a retrospective record review; the second case, occurring in 2013, was detected prospectively through active surveillance. Therefore, since the 2012 literature review (6), a total of 20 additional PADH cases have been identified, 18 cases in the literature and two cases in the United States, which are described as follows.

## Case Reports

### Patient A

In November 2008, a woman aged 49 years was diagnosed with severe falciparum malaria with 9% infected red blood cells, which is hyperparasitemia (defined as parasitemia >5%). She was successfully treated with parenteral artesunate and discharged from the hospital 4 days after admission. Her hemoglobin level was 11.1 g/dL at the time of admission and declined to 8.2 g/dL at the time of discharge. Eleven days after treatment initiation, the patient reported weakness, fatigue, shortness of breath, and bilateral lower extremity edema. On that date, her hemoglobin level was 5.7 g/dL; other laboratory test results included LDH 981 U/L, total bilirubin 1.2 mg/dL (normal = 0.1–0.9 mg/dL), and reticulocyte count 12.3% (normal = 0.5–1.5%). No other hematologic evaluation was done. She was given 3 units of packed red blood cells; her hemoglobin level subsequently rose to 9.8 g/dL 19 days after she first received artesunate. One week after the transfusion, the patient reported resolution of both shortness of breath and edema.

### Patient B

In August 2013, a man aged 26 years went to an outpatient clinic with a febrile illness that was presumptively treated as malaria. He received 2 doses of artemether-lumefantrine as an outpatient, but developed progressive confusion and went to the emergency department on the next day. That same day, he was admitted and diagnosed with severe falciparum malaria with >10% parasitemia; parenteral artesunate treatment was initiated. His hemoglobin level on admission was 8.9 g/dL, and he was transfused with 3 units of packed red bloods cells during the first 4 days of hospitalization. After the standard 3-day course of atovaquone-proguanil following artesunate therapy, his treatment with atovaquone-proguanil was extended to 8 days, longer than recommended by CDC’s malaria treatment guidelines and the artesunate IND protocol (9). His hemoglobin levels stabilized at 8.5 g/dL for several days. However, 9 days after treatment initiation with artesunate, his hemoglobin level dropped to 6.8 g/dL, and his total bilirubin rose to 3.4 mg/dL. Additional laboratory tests were consistent with acute hemolytic anemia: haptoglobin <0.1 g/L (normal = 0.2–2.0 g/L), LDH 5,882 U/L, and reticulocyte count 3.8%. The patient received 4 additional units of packed red blood cells and was discharged 13 days after parenteral artesunate initiation.

### Discussion

PADH can be a clinically relevant side effect after parenteral artesunate treatment. Follow-up visits up to 1 month following treatment with parenteral artesunate are recommended to detect and manage this condition. According to a proposed case definition, PADH is a nonrecurring event characterized by a 10% or greater decrease in hemoglobin levels in the setting of a haptoglobin level <0.1 g/L and an increase of LDH levels to >390 U/L, or an increase of ≥10% over baseline at least 7 days after initiation of parenteral artesunate treatment (10). Applying this case definition could help to improve specificity of PADH diagnoses. For example, considering the 19 cases from the 2012 literature review, the 18 identified by the recent literature review since then, and the two U. S. cases reported (39 cases in total), 23 had sufficient information and met the proposed case definition, one could be excluded from a PADH diagnosis, and 15 had insufficient information to assess exclusion, and should be considered as likely PADH cases (6–8).

Because all previously reported cases were treated with parenteral artesunate produced by facilities not accredited for good manufacturing practice (GMP) standards, direct toxicity resulting from treatment with non-GMP parenteral artesunate had been hypothesized as a PADH cause (6). The recent occurrence of two PADH cases in the United States, where only GMP-assured artesunate is used, conflicts with this hypothesis. A recently published report observed that PADH is more likely to occur in patients with high parasitemia (8). In contrast to treatment with quinine, which does result in immediate destruction of infected erythrocytes, artesunate kills malaria parasites, which are then selectively removed from erythrocytes by the spleen without immediate erythrocyte destruction. Following completion of artesunate treatment, these once-infected erythrocytes continue to circulate, but with a reduced life span. Once-infected erythrocytes are removed by the spleen at the end of their lifespan, 1–3 weeks. This event happens rather synchronously, and a substantial increase in hemolysis occurs, with the resultant decrease in hemoglobin levels (7,8). The report of 60 French returned travelers from countries where malaria is endemic found that the 13 with PADH had higher concentrations of once-infected erythrocytes compared with those diagnosed with non-PADH anemia 1 week after parenteral artesunate treatment initiation. In addition, higher parasitemia was associated with higher concentrations of once-infected erythrocytes, and the level of once-infected erythrocytes was a strong predictor of PADH (8).

Flow cytometry, which is available at most modern hospitals, can be adapted to estimate once-infected erythrocyte levels. The development of such a point-of-care test that can identify patients at risk for PADH with a high sensitivity and specificity would be useful in identifying those patients who would benefit from more stringent follow-up (8). Until the development and implementation of such a point-of-care test, all patients treated with parenteral artesunate should continue to be followed up for 1 month after treatment.

What is already known on this topic?

Parenteral artesunate, an artemisinin that is a first-line treatment for severe malaria in many countries, is associated with increased survival and has a better safety profile than the parenteral alternatives, quinine and quinidine. However, post-artemisinin delayed hemolysis (PADH) can occur 1–3 weeks after initiation of malaria treatment with drugs in the artemisinin class of antimalarials, resulting in anemia. The incidence of PADH is not precisely known.

What is added by this report?

In addition to 18 new PADH cases identified through a literature review, one U.S. case was identified through retrospective review and one through active surveillance, for a total of 20 new PADH cases. It is unlikely that the two U.S. PADH cases, in patients treated with artesunate meeting good manufacturing practices, resulted from drug toxicity related to manufacturing practices. Instead, hemolysis probably resulted from delayed clearance of once-infected erythrocytes, which continue to circulate after artesunate has killed the parasites.

What are the implications for public health practice?

Parenteral artesunate is a safe treatment for severe malaria.

PADH likely is the result of the delayed clearance of once-infected erythrocytes, which continue to circulate after the pharmacologic effect of parenteral artesunate, and is not the result of any toxic effect of parenteral artesunate. Therefore, parenteral artesunate is still considered safe and can remain as the first-line treatment for severe malaria in countries where it is licensed for use. Parenteral artesunate in the United States is available to qualifying patients by contacting CDC.*

## Footnotes

* At telephone 770-488-7788, Monday through Friday, from 9 a.m. to 5 p.m., or at 770-488-7100, after hours.
